# Population pharmacokinetics of rilpivirine following oral administration and long-acting intramuscular injection in real-world people with HIV

**DOI:** 10.3389/fphar.2024.1437400

**Published:** 2024-11-15

**Authors:** Paul Thoueille, Susana Alves Saldanha, Fabian Schaller, Eva Choong, François Veuve, Aline Munting, Matthias Cavassini, Dominique Braun, Huldrych F. Günthard, Jessy J. Duran Ramirez, Bernard Surial, Hansjakob Furrer, Andri Rauch, Pilar Ustero, Alexandra Calmy, Marcel Stöckle, Caroline Di Benedetto, Enos Bernasconi, Patrick Schmid, Catia Marzolini, François R. Girardin, Thierry Buclin, Laurent A. Decosterd, Monia Guidi

**Affiliations:** ^1^ Service of Clinical Pharmacology, Department of Medicine, Lausanne University Hospital and University of Lausanne, Lausanne, Switzerland; ^2^ Laboratory of Clinical Pharmacology, Department of Laboratory Medicine and Pathology, Lausanne University Hospital and University of Lausanne, Lausanne, Switzerland; ^3^ Service of Infectious Diseases, Department of Medicine, Lausanne University Hospital and University of Lausanne, Lausanne, Switzerland; ^4^ Department of Infectious Diseases and Hospital Epidemiology, University Hospital Zurich, Zurich, Switzerland; ^5^ Institute of Medical Virology, University of Zurich, Zurich, Switzerland; ^6^ Department of Infectious Diseases, Inselspital, Bern University Hospital, University of Bern, Bern, Switzerland; ^7^ Division of Infectious Diseases, Geneva University Hospitals, Faculty of Medicine, Geneva, Switzerland; ^8^ Department of Medicine, Faculty of Medicine, University of Geneva, Geneva, Switzerland; ^9^ Division of Infectious Diseases and Hospital Epidemiology, University Hospital Basel, University of Basel, Basel, Switzerland; ^10^ Division of Infectious Diseases, Ente Ospedaliero Cantonale, Lugano, Switzerland; ^11^ Division of Infectious diseases, Ente Ospedaliero Cantonale, University of Geneva, and University of Southern Switzerland, Lugano, Switzerland; ^12^ Division of Infectious Diseases and Hospital Epidemiology, Cantonal Hospital St Gallen, St Gallen, Switzerland; ^13^ Department of Molecular and Clinical Pharmacology, Institute of Translational Medicine, University of Liverpool, Liverpool, United Kingdom; ^14^ Centre for Research and Innovation in Clinical Pharmaceutical Sciences, Lausanne University Hospital and University of Lausanne, Lausanne, Switzerland; ^15^ Institute of Pharmaceutical Sciences of Western Switzerland, University of Geneva, University of Lausanne, Geneva, Switzerland

**Keywords:** rilpivirine, population pharmacokinetics, HIV, NONMEM, long-acting injectable

## Abstract

**Background:**

The pharmacokinetics of long-acting rilpivirine has mostly been studied in clinical trials, which do not fully address the uncertainties that arise in routine clinical situations.

**Aims and methods:**

Our population analysis aims to establish percentile curves for rilpivirine concentrations in people with HIV (PWH) followed-up in a routine clinical setting, while identifying patient-related factors that may influence rilpivirine exposure. A total of 238 PWH enrolled in our nationwide multicenter observational study contributed to 1038 concentrations (186 and 852 concentrations after oral and intramuscular injection, respectively).

**Results:**

Rilpivirine pharmacokinetics were best described by a two-compartment model with an oral to intramuscular relative bioavailability factor. A simple zero-order absorption process was retained for oral administration while a parallel first-order absorption was used for intramuscular administration, with 27.6% of the dose released via a fast absorption pathway and the remaining fraction via a slow absorption pathway. Our model estimated that long-acting rilpivirine reaches steady-state after 2.5 years and has an elimination half-life of 18 weeks, consistent with published estimates. In females, a 45.6% reduction in the proportion of the dose absorbed via the rapid absorption pathway was observed. However, this resulted in no more than 15% difference in trough concentrations (C_trough_) compared to males, which was not considered to be clinically relevant.

**Conclusion:**

Overall, our model-based simulations showed that only approximately 50% of long-acting rilpivirine C_trough_ would be above the 50 ng/mL threshold associated with optimal therapeutic response, while approximately 85% of C_trough_ would be above the first quartile of concentrations observed in Phase III trials (32 ng/mL).

## 1 Introduction

Rilpivirine is a non-nucleoside reverse transcriptase inhibitor (NNRTI) prescribed orally in combination with emtricitabine and tenofovir in treatment-naïve patients with a viral load below 100,000 copies at baseline (U.S. Food and Drug Administration, 2011). In addition, rilpivirine, in combination with dolutegravir, is usually prescribed as a complete regimen for people whose viral load has remained below 50 copies/mL for at least 3 months (U.S. Food and Drug Administration, 2021). Recently, rilpivirine was formulated as a nanosuspension for intramuscular (i.m.) injection, which allowed the apparent elimination half-life (t_1/2_) of rilpivirine to be extended from 45 h to approximately 13–28 weeks (Hodge et al., 2021; U.S. Food and Drug Administration, 2022; European Medicines Agency (EMA), 2021a). Rilpivirine in combination with cabotegravir is the first long-acting regimen for the maintenance treatment of HIV-1 infection in adults (U.S. Food and Drug Administration, 2022; European Medicines Agency (EMA), 2021a; European Medicines Agency (EMA), 2021b). Following an oral initiation period, an i.m. injection of 900 mg of rilpivirine *plus* 600 mg of cabotegravir is administered into the gluteal muscle. Subsequently, 900 mg of rilpivirine *plus* 600 mg of cabotegravir is given i.m. every 2 months, or monthly in some regions with 600 mg of rilpivirine *plus* 400 mg of cabotegravir.

Population pharmacokinetic (popPK) analysis of long-acting rilpivirine based on data from Phase III registrational trials suggested a one-compartmental model with linear elimination and two parallel sequential zero-first-order absorption pathways to describe the drug pharmacokinetics (Neyens et al., 2021; Benaboud et al., 2023). The analysis showed that rilpivirine concentrations following i.m. injection and oral administration were similar. In addition, no demographic or clinical covariates were found to significantly impact rilpivirine pharmacokinetics. Regarding therapeutic plasma exposure, the protein-adjusted concentration required for 90% inhibition of viral replication (PAIC_90_) with rilpivirine is 12 ng/mL (Margolis et al., 2015). However, the clinical target thresholds are higher based on available drug exposure–response studies. A threshold of 50 ng/mL has been recommended as the minimum concentration to increase the likelihood of a therapeutic response (Aouri et al., 2016; Dickinson et al., 2015). Some authors concluded that even higher plasma levels of rilpivirine of up to 100 ng/mL should be targeted (Néant et al., 2019). Finally, multivariable analyses showed that rilpivirine resistance mutations at baseline, HIV-1 subtype A6/A1, body mass index (BMI) greater than 30 kg/m^2^, or low rilpivirine and/or cabotegravir trough concentration (C_trough_) 4 weeks after the initial loading dose (i.e., 32 ng/mL and 1,120 ng/mL, respectively, corresponding to the first quartile of concentrations observed in Phase III trials) were associated with an increased risk of virologic failure (European Medicines Agency (EMA), 2021a; European Medicines Agency (EMA), 2021b; Cutrell et al., 2021; Orkin et al., 2023).

This popPK analysis is part of a Swiss observational study designed to monitor drug levels in people with HIV (PWH) who are treated with long-acting injectable cabotegravir *plus* rilpivirine (Thoueille et al., 2024a). The present study provides the first description of long-acting rilpivirine concentration–time profiles and their variability in PWH in a routine clinical setting and aims to establish percentile curves to help with the interpretation of drug concentration measurements as part of therapeutic drug monitoring (Thoueille et al., 2024b). Cabotegravir reference curves are described in a separate article for clarity (Thoueille et al., 2024c).

## 2 Methods

### 2.1 Study population

The Swiss HIV Cohort Study (SHCS) was established in 1988 and is a prospective longitudinal study for the follow-up of PWH (>18 years old) in Switzerland (Scherrer et al., 2022). Written informed consent was obtained from all participants, and the SHCS was approved by Canton Ethics Committees. The majority of drug samples were collected at the discretion of physicians longitudinally (i.e., sparse samples) from March 2022 to June 2023, following the approval of long-acting cabotegravir *plus* rilpivirine in Switzerland. All PWH enrolled in the nationwide observational study were considered for the popPK analysis (Thoueille et al., 2024a). In addition, a detailed sampling plan within a dosing interval was offered to consenting PWH followed-up in Lausanne and Geneva (Switzerland) receiving long-acting rilpivirine. This substudy consisted of blood sampling taken before the dose injections and at 1 week, 2 weeks, 4 weeks, and 8 weeks (corresponding to the end of the dosing interval, C_trough_) after the i.m. dose (Project-ID 2022-00619, approved by the Canton’s Ethics Committee, Lausanne, Switzerland).

The dose of rilpivirine, timing of the blood sampling and the last dose, bodyweight (BW), height, and body mass index (BMI) were recorded. Additional clinical and demographic information, such as sex at birth, age, viremia, CD4 cell count, and co-medications were extracted from the SHCS database. The criteria for exclusion from our popPK analysis included one undetectable rilpivirine plasma concentration after oral administration, which was due to non-adherence to oral treatment. In addition, observations were excluded if there was unreliable information about the time and/or date of the last drug administration and/or blood collection.

### 2.2 Analytical method

Samples were analyzed by a previously published validated multiplex high-performance liquid chromatography coupled to tandem mass spectrometry with a lower limit of quantification of 5 ng/mL (Courlet et al., 2020).

### 2.3 Population pharmacokinetic analysis

The non-linear mixed effects modeling was performed using the software NONMEM^®^ (v7.5.1, ICON Development Solutions, Ellicott City, MD, USA), assisted by PsN (v5.3.1) and Pirana (v2.9.3). Data management, visual exploration, and statistical analyses were performed using R (v4.1.1, R Development Core Team, http://www.r-project.org/). As samples were collected at least 2 weeks after treatment initiation, steady-state levels were assumed for all samples collected during the oral lead-in period (i.e., oral rilpivirine half-life (t_1/2_) of 45–50 h) (Rilpivirine (oral) PK Fact Sheet, 2021). It should be noted that most PWH contributed to only one sample for oral rilpivirine, as samples were conveniently collected immediately before the i.m. loading dose (i.e., few samples were collected after oral administration). Regarding i.m. injections, steady-state levels were assumed from week 96 in accordance with available information (Overton et al., 2023). Two PWH received long-acting cabotegravir and rilpivirine every 4 weeks (400 mg/600 mg) for compassionate use before Swiss market authorization. This was the only recommended regimen at that time. These PWH were switched to the two-monthly regimen (600 mg/900 mg of cabotegravir/rilpivirine) a few months after the start of the study.

### 2.4 Model building and selection

A classic stepwise procedure was used to identify the model that best fitted the concentrations of rilpivirine after oral and i.m. administrations. The models were specified through differential equations using the NONMEM^®^ subroutine ADVAN13 to best depict the dynamics of the longitudinal data collected. One- and two-compartment models with different absorption processes and linear elimination were compared. During preliminary model developments, the use of a zero-order absorption ( $D_{oral}$ ), rather than a first-order absorption, was found to best describe the concentrations after oral administration. However, because of the limited number of samples collected right after drug oral intake, $D_{oral}$ was fixed to 4 h in accordance with available information and our preliminary estimation (U.S. Food and Drug Administration, 2011; Aouri et al., 2016). Between-subject variability (BSV) was sequentially tested on all the parameters, which were assumed to follow log-normal distributions.

The absorption process of long-acting rilpivirine was best described by a parallel first-order absorption ( $k_{a_{fast}}$ and $k_{a_{slow}}$ ), with a fraction of the dose ( $F_{i.m.fast}$ ) released via a relatively fast absorption pathway constrained between 0 and 1 using the following equation (Bouzom et al., 2000):

$$TEMP=\mathrm{Ln}\left[\frac{\theta_{F_{i.m.fast}}}{1-\theta_{F_{i.m.fast}}}\right]$$

$$F_{{i.m.fast}_{i}}=\frac{\exp\;\left(TEMP+\eta_{i}^{F_{i.m.fast}}\right)}{1+\exp\;\left(TEMP+\eta_{i}^{F_{i.m.fast}}\right)}$$

where TEMP is a temporary variable allowing estimation of $F_{i.m.fast}$ , $\theta_{F_{i.m.fast}}$ is the typical value of $F_{i.m.fast}$ in our population, $F_{{i.m.fast}_{i}}$ is the estimated $F_{i.m.fast}$ in the *i* ^th^ individual, and $\eta_{i}^{F_{i.m.fast}}$ corresponds to the BSV term. The remaining fraction of the dose was released via a slow absorption pathway ( $F_{{i.m.slow}_{i}}=1-F_{{i.m.fast}_{i}}$ ). In order to account for within-subject variability between injections (such as unexplained physiological differences or injection-related variability), inter-occasion variability (IOV) was also considered. In particular, occasions were coded to be consistent with the duration of the follow-up by including an “occasion” variable constructed with an incremental number within each subject for a maximum of six occasions/injections. Lastly, common and distinct residual unexplained variabilities (RUV) were evaluated for oral and i.m. administration. The following covariates were tested for significance on the base model parameters using linear functions: sex at birth, age, ethnicity, BW, BMI, and eGFR categories [classified according to the CKD-EPI equations (Levey et al., 2009)]. BW and BMI were also evaluated using allometric scaling relationships to estimate the allometric exponent. No clinically relevant concomitant drugs, such as potent 3A4 inducers, were encountered in the study population (European Medicines Agency (EMA), 2021a).

The variation of the NONMEM^®^ objective function value (ΔOFV) was used at a 0.05 significance level in the forward model-building step (ΔOFV < −3.84 for one additional parameter) to statistically discriminate hierarchical models. During the backward deletion step, a significance level of 0.01 (ΔOFV >6.63 for the removal of one parameter) was used. Non-nested models were discriminated using Akaike’s information criterion. Model selection relied on diagnostic plots and the accuracy of PK parameter estimates, quantified by the relative standard error (RSE). The reliability of the results was also assessed by characterizing model shrinkage and the normality of the distribution of individual eta estimates.

### 2.5 Model-based Monte Carlo simulations

The clinical relevance of covariates was evaluated by comparing the PK profiles and C_trough_ values of rilpivirine obtained in different groups of interest. Population percentiles for rilpivirine after oral and i.m. administration were generated to help interpret drug concentration measurements as part of therapeutic drug monitoring.

### 2.6 Model validation

The observed concentrations were compared with the 5th, 50th, and 95th prediction percentiles using prediction-corrected visual predictive checks (pcVPCs) performed on the final popPK model (Lindbom et al., 2005; Bergstrand et al., 2011; Jonsson and Karlsson, 1999). In addition, the original model estimates were examined against the bootstrap median parameter values and their 95% confidence intervals generated using 2000 replicates (Lindbom et al., 2005). Finally, cross-validation was performed using repeated data-splitting (n = 5) to create random subsets of the dataset, with 80% allocated to the modeling dataset and 20% to the validation dataset. Log-transformed individual observed and predicted concentrations were then evaluated using mean prediction error (MPE) and root mean square error (RMSE) as metrics to assess model accuracy and precision, respectively (Sheiner and Beal, 1981).

## 3 Results

Overall, 238 PWH contributed to 1,038 rilpivirine concentrations (186 concentrations after oral administration and 852 concentrations after i.m. injection collected from 176 PWH and 222 PWH, respectively), with detailed PK investigation performed on 28 PWH. Table 1 summarizes the characteristics of the PWH included in the analysis. Overall, four samples (range: 1–15) were collected per individual, with one (1–3) for oral rilpivirine and three (1–14) for i.m. rilpivirine. The median duration of follow-up was 26 weeks (3–196). Only 10 PWH had rilpivirine concentrations assumed to be at a steady state (i.e., from week 96) after i.m. administration.

**TABLE 1:** Characteristics of the PWH.

Population characteristics Last recorded value	Median (range) or n (%)
Sex Male Female	190 (80) 48 (20)
Age, years	46 (20–79)
Ethnicity White Black Hispanic American Asian Other/Missing	133 (56) 36 (15) 19 (8) 11 (5) 39 (16)
Body weight, kg	78 (50–126)
Height, cm	176 (151–198)
BMI, kg/m^2^ < 25 25–30 >30	25.4 (18.2–43.3) 104 (44) 103 (43) 31 (13)
eGFR^a^, mL/min/1.73 m^2^ (Levey et al., 2009) G1: ≥90 G2: 60–89 G3: 30–59	158 (66) 76 (32) 4 (2)
Liver cirrhosis^a^, Child–Pugh score (Child and Turcotte, 1964) No Class A	236 (99) 2 (1)
CD4 cell count, cells/mm^3^ ≥ 500 350 to <500 <350	186 (78) 23 (10) 29 (12)
Plasma HIV RNA, copies/mL < 50 ≥ 50 and < 200 ≥ 200	233 (98) 4 (2) 1 (<1)

BMI, body mass index; eGFR: estimated glomerular filtration rate, calculated according to the CKD-EPI equations reported by Levey et al. (2009).

^a^ ≤2% missing information.

### 3.1 Structural and covariate models

**FIGURE 1:**
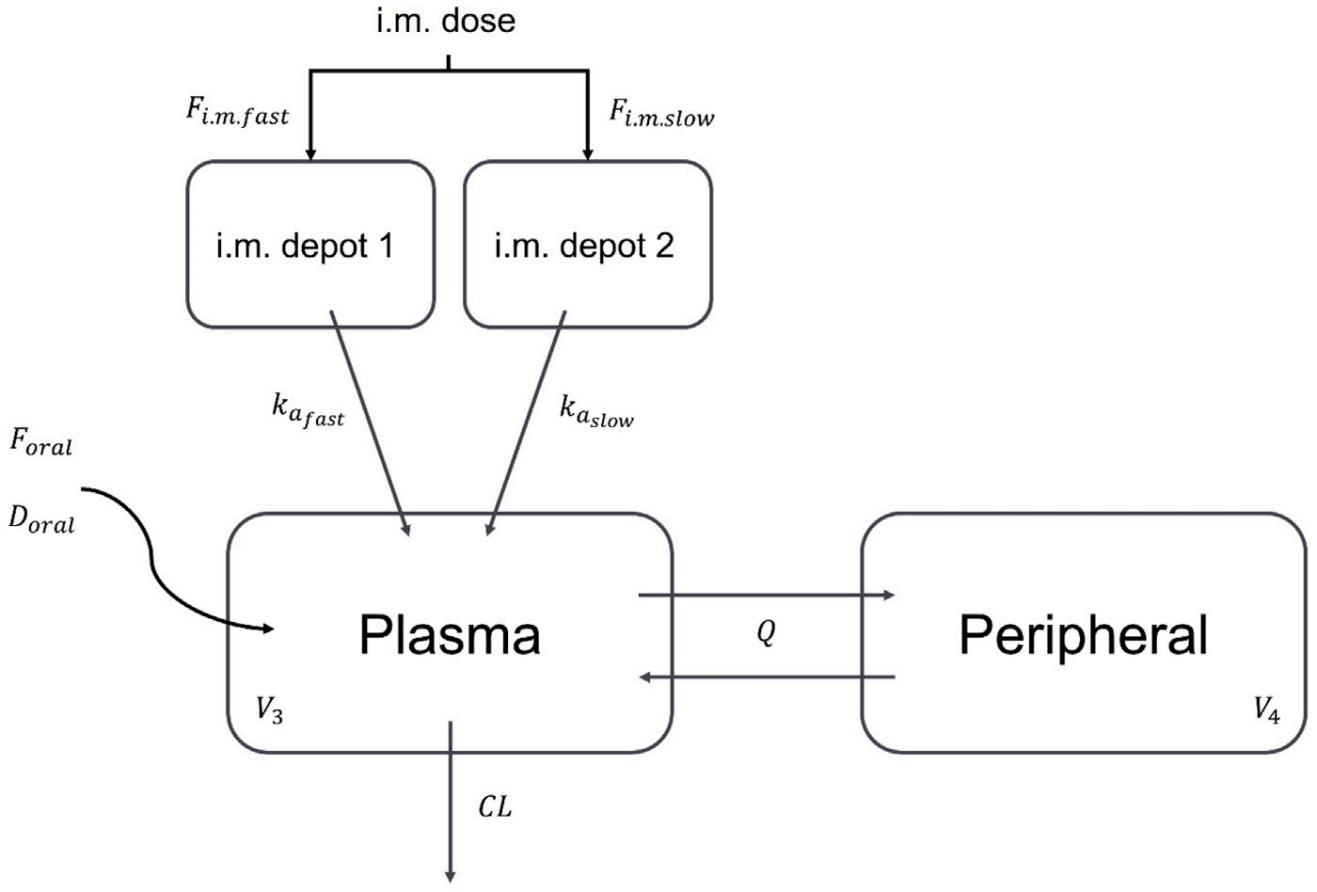
Structural model used to describe rilpivirine concentration-time profile. i.m.: intramuscular; $F_{oral}$ : absolute bioavailability of oral to i.m. administrations; $D_{oral}$ : zero-order absorption time for oral administration; $F_{i.m.fast}$ : fraction for the fast absorption pathway after i.m. administration; $k_{a_{fast}}$ and $k_{a_{slow}}$ : fast and slow first-order absorption rate constants for i.m. administration, respectively; $V_{3}$ : apparent central volume of distribution; Q : apparent intercompartmental clearance; $V_{4}$ : apparent peripheral volume of distribution; CL : apparent clearance.

Consistent with available information (Neyens et al., 2021), long-acting rilpivirine was characterized by “flip-flop” kinetics (i.e., absorption rate constants ( $k_{a_{fast}}$ and $k_{a_{slow}}$ ) lower than elimination rate constant ( $k_{e}=CL/V$ )). Figure 1 presents the structural model that best described rilpivirine concentrations. The absorption process of long-acting rilpivirine was best described by a parallel first-order absorption (ΔOFV = −192, *p* < 0.001, compared to the model with a single first-order absorption). On the other hand, a zero-order absorption process ( $D_{oral}$ , fixed to literature value) was retained for oral administration, with the inclusion of a relative bioavailability ( $F_{oral}$ ) (i.e., as i.m. administration was assumed to be 100% bioavailable) with BSV (ΔOFV = −15, *p* < 0.001). In addition, it was found that a two-compartment model (with $V_{3}$ and $V_{4}$ volumes of distribution of the central and peripheral compartments, respectively) provided the best description of the data (ΔOFV = −20, *p* < 0.001). The assignment of BSV on $k_{a_{slow}}$ significantly improved data description (ΔOFV = −48, *p* < 0.001), while the IOV after i.m. administration was supported for clearance ( CL ) (ΔOFV = −32, *p* < 0.001). Importantly, we investigated the inclusion of IOV in the absorption process of i.m. rilpivirine. However, such variability could not be retained in the absorption parameters due to model stability and statistical significance. Finally, a common mixed error model for both routes of administration failed to estimate both components of the error model. An additive error model best described rilpivirine RUV after oral administration, while a proportional error model was retained for rilpivirine RUV when administered i.m. Parameter estimates of the base popPK model with BSV (CV%) were: a $D_{oral}$ of 4 h (fixed), an $F_{oral}$ of 65.4% (37.1%), a $k_{a_{fast}}$ of 0.00214 h^−1^ with an $F_{i.m.fast}$ of 27.6% (16.8%), a $k_{a_{slow}}$ of 0.000229 h^−1^ (82.7%), a $V_{3}$ of 277 L, an inter-compartment clearance ( Q ) of 4.08 L/h, a $V_{4}$ of 839 L, and a CL of 6.74 L/h (25.9%) with an IOV of 13%.

Univariate analyses revealed the effects of female sex (ΔOFV = −11, *p* < 0.001), and BMI (ΔOFV = −6, *p* < 0.05) on $F_{i.m.fast}$ . After forward insertion (*p* < 0.05) and backward deletion (*p* < 0.01), the model only included the effect of female sex as a percent change on $F_{i.m.fast}$ :

$$TEMP=\mathrm{Ln}\left[\frac{\theta_{F_{i.m.fast}}\cdot \left(1+\theta_{Female}\right)}{1-\theta_{F_{i.m.fast}}\cdot \left(1+\theta_{Female}\right)}\right]$$

This effect showed that females had an $F_{i.m.fast}$ 45.6% lower than males, resulting in a greater proportion of the dose absorbed via the slow absorption pathway. Finally, the sex covariate explained 11% of the BSV on $F_{i.m.fast}$ .

### 3.2 Model-based Monte Carlo simulations

**FIGURE 2:**
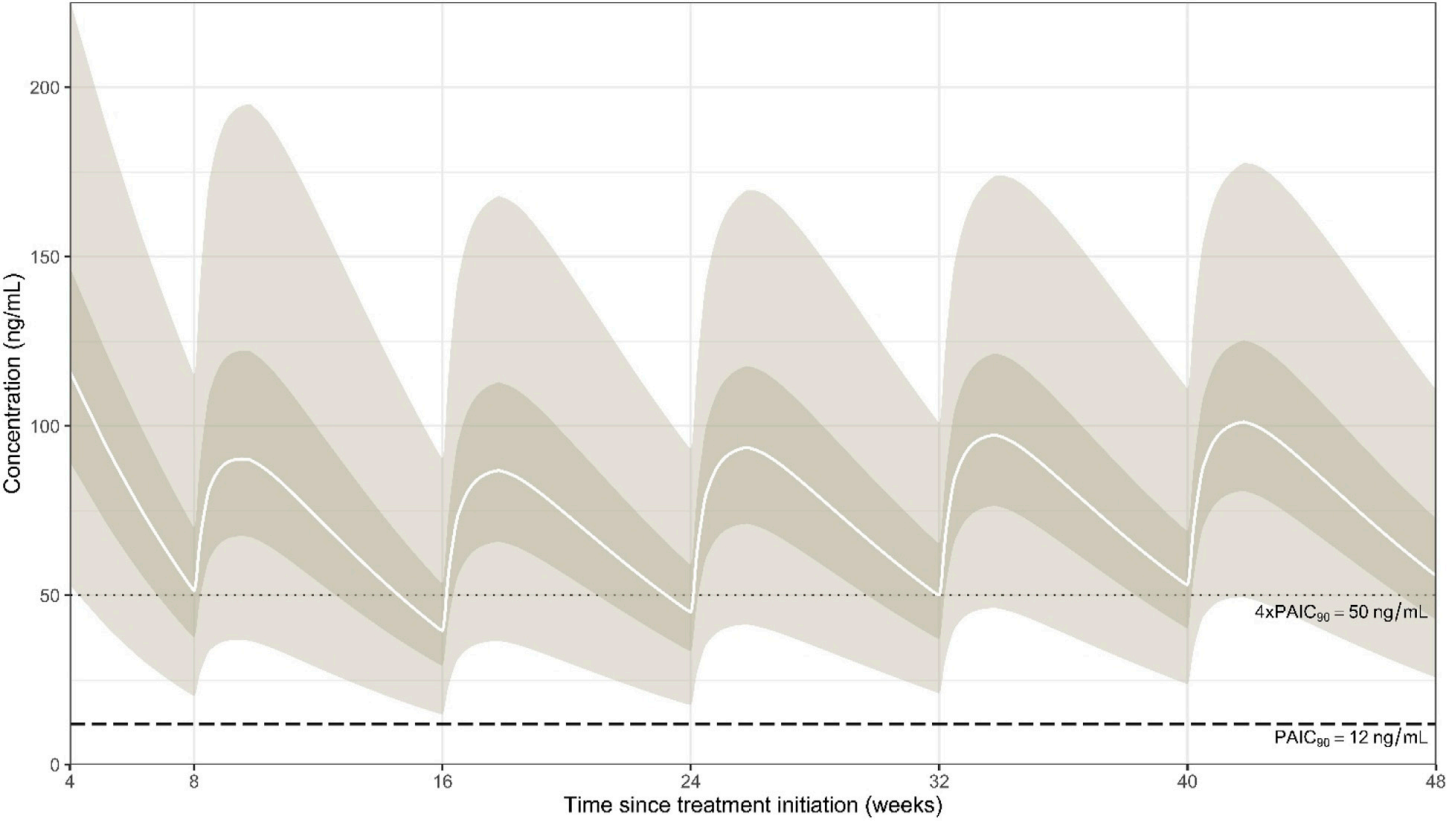
Simulated population percentiles after intramuscular administration of rilpivirine following a 4-week period of oral lead-in. The solid white lines represent the median (50% percentile), while the dark surfaces encompass the 50% prediction intervals, and the light surfaces encompass the 95% prediction intervals. The horizontal dashed line represents the PAIC_90_ of 12 ng/mL, while the dotted line shows the threshold of 50 ng/mL.

Supplementary Figure S2 shows the PK profile with variability obtained for rilpivirine after oral administration. The median C_trough_ at a steady state under oral rilpivirine was 80 ng/mL [95% prediction interval (PI_95_): 32–211]. Our model-based simulations suggest that 85% of PWH receiving oral rilpivirine would have a C_trough_ above the target of 50 ng/mL associated with therapeutic response. Regarding long-acting rilpivirine, it was found that females had a 12% lower rilpivirine C_trough_ 4 weeks after the loading dose (Supplementary Figures S3, S4; Supplementary Table S2). Then, females had 8%, 12%, 10%, 14%, and 15% higher rilpivirine C_trough_ at weeks 16, 24, 32, 40, and 48, respectively. Therefore, although statistically significant, the effect of sex on long-acting rilpivirine C_trough_ was not considered clinically relevant, and this model was not validated. Indeed, the base and covariate models showed a maximum difference of 14% in the prediction of i.m. rilpivirine C_trough_ (Supplementary Table S2). Figure 2 presents the population PK profile obtained for rilpivirine after i.m. injection (i.e., without any covariate). Overall, our model-based simulations showed that only 50% of rilpvirine C_trough_ values were above the 50 ng/mL threshold after i.m. injection at week 8 (i.e., 4 weeks after the loading dose). A median 22% reduction in C_trough_ values was observed at week 16. Subsequently, rilpivirine C_trough_ values were found to increase through week 48 but remained almost 50% below the 50 ng/mL target at all time points. Finally, model-based simulations suggested that approximately 5% and almost 15% of PWH would have C_trough_ values below 2×PAIC_90_ (i.e., 24 ng/mL) and below the first quartile of concentrations observed in Phase III trials (i.e., 32 ng/mL), respectively.

### 3.3 Model validation

**FIGURE 3:**
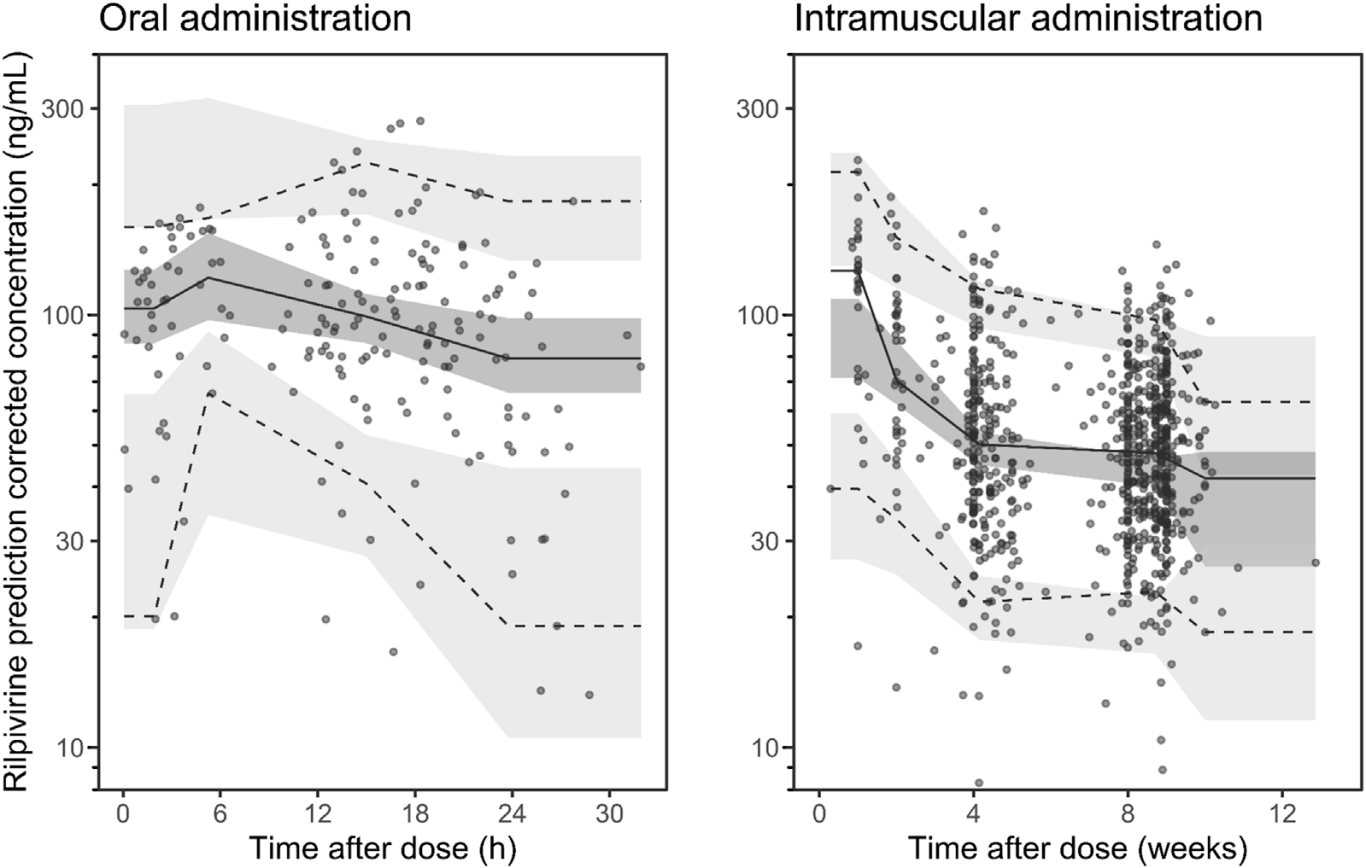
Visual predictive check of the retained rilpivirine popPK model for oral administration (left panel) and for intramuscular administration (right panel) for rilpivirine. Open circles represent the observed plasma concentrations. Solid and dashed lines represent the median and 90% prediction intervals (PI_90%_) of the observed data, respectively. Dark- and light-shaded surfaces represent the model-predicted 90% confidence intervals of the simulated median and PI_90%_. Note: one concentration with time beyond 4000 h is not displayed.

Supplementary Figure S1 shows the goodness-of-fit diagnostic plots of the base model as no covariates were found to be clinically relevant. It should be noted that there was a modest shrinkage of 40% on the BSV of $F_{i.m.fast}$ , which could make the use of diagnostic plots of limited value (Savic and Karlsson, 2009). However, the pcVPC and the bootstrap results, shown in Figure 3 and Table 2, respectively, support the reliability of the final model (without covariate). Despite a modest model misspecification in the first days of LAI therapy, probably due to the paucity of data at the early stage of treatment, the pcVPC supports the adequacy of the model developed, particularly in predicting the trough concentrations essential for clinical decisions. Finally, cross-validation (Supplementary Table S1) revealed no significant bias (mean MPE = 0.7%, mean 95% confidence interval = −1.4% to 2.9%) with a precision of 16.8% (range: 14.3%–19.1%).

**TABLE 2:** Final population PK parameter estimates of rilpivirine with their bootstrap evaluations.

Parameters	Final model	Bootstrap (n = 2000)
	Estimate (RSE, %)^b^	Median [CI_95%_]
$F_{oral}$ (%)	65.4 (5)	64.8 [52.6–73.3]
$\omega_{F_{oral}}$ (CV%^a^)	37.1 (11)	36.8 [24.5–46.3]
$D_{oral}$ (h)	4 FIX	4 FIX
$F_{i.m.fast}$ (%)	27.6 (9)	27.5 [22.5–32.4]
$\omega_{F_{i.m.fast}}$ (CV%^c^)	16.8 (15) ^d^	16.7 [10.0–25.3]
$k_{a_{fast}}$ (h^−1^)	0.00214 (11)	0.00211 [0.00167–0.00266]
$k_{a_{slow}}$ (h^−1^)	0.000229 (11)	0.000225 [0.000108–0.000292]
$\omega_{k_{a_{slow}}}$ (CV%^a^)	82.7 (12)	80.6 [51.5–106.0]
$V_{3}$ (L)	277 (25)	274 [184–433]
Q (L/h)	4.08 (40)	4.03 [1.75–9.30]
$V_{4}$ (L)	839 (11)	853 [407–1,365]
CL (L/h)	6.74 (3)	6.68 [5.41–7.37]
$\omega_{CL}$ (CV%^a^)	25.9 (9)	25.5 [17.6–30.3]
$\omega_{IOV}$ (CV%^a^)	13.0 (10)	13.0 [5.35–17.6]
$\sigma_{add-oral}$ (ng/mL)	18 (22)	17.7 [10.5–28.1]
$\sigma_{prop-LA}$ (CV%^a^)	18 (5)	17.5 [15.2–20.1]

$F_{oral}$ : typical relative bioavailability of oral to i.m. administration; $D_{oral}$ : typical zero-order absorption time for oral administration; $F_{i.m.fast}$ : fraction for the fast absorption pathway after intramuscular administration; $k_{a_{fast}}$ and $k_{a_{slow}}$ : typical fast and slow first-order absorption rate constants for intramuscular administration, respectively; $V_{3}$ : typical apparent central volume of distribution; Q : typical apparent intercompartmental clearance; $V_{4}$ : typical apparent peripheral volume of distribution; CL : typical apparent clearance; ω : between-subject variability (BSV); $\omega_{IOV}$ : inter-occasion variability (IOV); $\sigma_{add-oral}$ : additive residual error for oral administration; $\sigma_{prop-LA}$ : proportional residual error for intramuscular administration.

^a^ Coefficient of variation (CV, %) for BSV was calculated as follows: $CV=\sqrt{\left(e^{\omega^{2}}-1\right)}$

^b^ Relative standard error (RSE) of the estimate, expressed as a percentage, with standard error (SE) of estimate, calculated as follows: $RSE\;\left(\%\right)=\frac{e^{\omega^{2}}\cdot SE}{2\cdot \left(e^{\omega^{2}}-1\right)}$

^c^ Coefficient of variation (CV, %) for the BSV of $F_{i.m.fast}$ was approximated as: ${CV}_{F_{i.m.fast}}=\theta_{F_{i.m.fast}}\cdot \left(1-\theta_{F_{i.m.fast}}\right)\cdot \sqrt{\eta^{F_{i.m.fast}}}$

^d^ Calculated using the propagation error formula.

## 4 Discussion

The present study describes the first popPK model of rilpivirine in PWH followed in a routine clinical setting, including both oral and i.m. routes of administration. The parallel absorption process found in our popPK model for long-acting rilpivirine is consistent with a previously published popPK model based on Phase III registrational studies (Neyens et al., 2021). Our model shows that approximately a quarter (i.e., 27.6%) of the nanosuspension dose is absorbed via a relatively fast absorption pathway, while the remaining (i.e., 72.4%) is absorbed via a ten-time slower absorption pathway. Females were found to have an almost 50% reduction in the fraction of the dose absorbed via the fast absorption pathway, thereby having overall slower drug absorption than males. Because of the “flip-flop” kinetics displayed by long-acting rilpivirine, this slower absorption resulted in lower concentration in females at week 8 (i.e., 4 weeks after the loading dose). Females then exhibited higher concentrations from week 16 and onward. However, as there were no more than a 15% difference in rilpivirine C_trough_ levels throughout 48 weeks and the addition of this covariate explained only 11% of the BSV on $F_{i.m.fast}$ , the effect of sex was not considered clinically relevant and was thus not retained in the final validated model.

On another note, preliminary model developments showed that the clearance of rilpivirine after i.m. injection was 18% lower than the clearance calculated for oral rilpivirine (with distinct BSV). In addition to being statistically significant, an estimation of the relative bioavailability ( $F_{\text{oral}}$ ) with BSV of oral to i.m. rilpivirine was considered appropriate because it plausibly reflects the biological variability affecting rilpivirine concentrations using the different routes of administration. Indeed, $F_{\text{oral}}$ allows correcting for rilpivirine dose, accounts for BSV, and thus also impacts the apparent PK parameters for oral rilpivirine. In particular, it was found that without the introduction of $F_{\text{oral}}$ into the model, the central volume of distribution estimate was markedly increased (approximately 3000 L), resulting in biased predictions of rilpivirine concentrations after oral administration. Previously published popPK analyses performed on oral rilpivirine found central volumes of distribution of 321 and 401 L to describe the disposition of rilpivirine (Aouri et al., 2016; Néant et al., 2018), which is consistent with our preliminary development results on oral rilpivirine data showing that a one-compartment model with a central volume of 409 L provided the best model fit. However, because rilpivirine is a highly lipophilic drug (log *p* = 4.86), it is distributed widely into tissue compartments (Eke et al., 2018). The data collected after i.m. injection probably helped describe the peripheral compartment found in our final model, which included both routes of administration. Lastly, our popPK model shows significant BSV on PK parameters and a modest IOV on CL , which have not been described before. The inclusion of IOV on the absorption process of long-acting rilpivirine was tested but not retained (i.e., based on statistical significance and model stability). Differences in rilpivirine absorption and/or resorption between occasion within subjects would seem appropriate (e.g., injection-related variability (Jucker et al., 2021), as well as physiological differences). However, in the absence of more informative data during the absorption phase, an IOV could not be estimated for the i.m. absorption. This probably also explains the modest shrinkage found on $F_{i.m.\text{fast}}$ BSV.

Our popPK model allowed the estimation of t_1/2_ for oral rilpivirine of 17 h and 240 h, corresponding to an initial decrease due to diffusion into the peripheral compartment and the onset of elimination and a final decrease due to elimination after equilibrium is reached, respectively. On the other hand, the time to reach a steady state and the t_1/2_ of long-acting rilpivirine were estimated using the absorption rate constant of the slowest absorption pathway ( $k_{a_{slow}}$ ). Our model shows that rilpivirine reaches steady-state levels after 1.7 [90% CI: 0.5–5.7] years and that the t_1/2_ is 18.0 [90%CI: 5.5–59.0] weeks. Although there is a wide range of variability, these values are consistent with those reported in the literature (European Medicines Agency (EMA), 2021a; Neyens et al., 2021).

Our model-based simulations showed that 15% of PWH taking oral rilpivirine would have a C_trough_ below the target of 50 ng/mL, which is twice lower than that previously reported by Aouri et al. (2016). We hypothesize that, because of the modality of drug administration (i.e., fasting condition because high gastric pH impairs intestinal absorption of rilpivirine (U.S. Food and Drug Administration, 2011) and the relatively short duration of the oral lead-in period, overall adherence in our study may have been better than in the PWH population of Aouri et al. (2016), which received oral rilpivirine in the long term. However, some of the low concentrations observed in PWH on oral rilpivirine might have been caused by impaired adherence. In addition, three PWH enrolled in our study had gastric bypass surgery, and their rilpivirine concentrations were found to be reduced after oral administration, as shown in Supplementary Figure S2 (Piso et al., 2023). This effect was not included in the model because statistical power prevented proper estimation of the PK parameters. Regarding long-acting rilpivirine, our model-based simulations showed that only approximately 50% of rilpivirine C_trough_ were above the 50 ng/mL threshold after i.m. injection. This finding is consistent with information from Phase III registrational studies (Cutrell et al., 2021; Overton et al., 2023; Orkin et al., 2021). The results of our analysis indicate that the threshold of 2×PAIC_90_ (24 ng/mL) may be a more appropriate target for long-acting rilpivirine C_trough_ levels than the 4×PAIC_90_ value (50 ng/mL) (Thoueille et al., 2024b). The latter may be overly alarmist and prompt healthcare providers to discontinue long-acting treatment unnecessarily. Nevertheless, it is important to note that viral failure is still observed in individuals with rilpivirine levels below 50 ng/mL, particularly in PWH who have additional risk factors. Lastly, simulations showed that 5% of PWH would have rilpivirine C_trough_ below 2×PAIC_90_ (24 ng/mL), which may be of concern.

Limitations of the present work should be acknowledged. Because the majority of PWH contributed to one sample and no detailed PK sampling was available for oral rilpivirine, the discrimination between BSV and RUV variabilities was limited for oral rilpivirine PK. In addition, because sex was considered clinically irrelevant, the model including this covariate was not validated. Therefore, the results of model-based simulations including sex should be interpreted with caution. Although our study found that no clinical covariate influenced rilpivirine disposition in our real-world cohort from Switzerland, available evidence derived from physiologically-based pharmacokinetic (PBPK) modeling showed that morbidly obese PWH could be at higher risk of presenting suboptimal C_trough_ levels (Bettonte et al., 2024a). Such an effect could not be identified in our study due to the absence of morbidly obese individuals in our population. In addition, another recent PBPK study showed a modest increase in the exposure of long-acting rilpivirine in older compared to younger individuals, which nevertheless does not warrant a dose adjustment (Bettonte et al., 2024b). Similarly, the small number of older people in our study population may have mitigated the effect of age on rilpivirine PK. Further research is warranted to confirm whether our findings are applicable to populations from other settings. In addition, the design of our study provided limited opportunities to collect early concentrations during the first few days after i.m. injection. The detailed PK investigations revealed variable concentration patterns in some individuals, with some having consistently low plasma concentrations as early as 1 week to 2 weeks after the injection. The underlying cause of these unusual PK profiles remains uncertain within the scope of our analysis. At this time, it remains unclear whether low plasma levels associated with identified or unidentified risk factors could compromise the therapeutic success of long-acting cabotegravir and rilpivirine (Orkin et al., 2023).

In conclusion, our study provides the first long-acting rilpivirine concentration-time profiles and their variability in PWH in a routine clinical setting. In accordance with previous results, no covariate was found to clinically influence rilpivirine disposition. A comprehensive discussion of the thresholds to be used in the clinical setting can be found elsewhere (Thoueille et al., 2024a; Thoueille et al., 2024b).

## 5 Group members of the Swiss HIV Cohort Study

I. Abela, K. Aebi-Popp, A. Anagnostopoulos, M. Battegay, E. Bernasconi, D. L. Braun, H. C. Bucher, A. Calmy, M. Cavassini, A. Ciuffi, G. Dollenmaier, M. Egger, L. Elzi, J. Fehr, J. Fellay, H. Furrer, C. A. Fux, H. F. Günthard (President of the SHCS), A. Hachfeld, D. Haerry (deputy of “Positive Council”), B. Hasse, H. H. Hirsch, M. Hoffmann, I. Hösli, M. Huber, D. Jackson-Perry (patient representatives), C. R. Kahlert (Chairman of the Mother & Child Substudy), L. Kaiser, O. Keiser, T. Klimkait, R. D. Kouyos, H. Kovari, K. Kusejko (Head of Data Centre), N. Labhardt, K. Leuzinger, B. Martinez de Tejada, C. Marzolini, K. J. Metzner, N. Müller, J. Nemeth, D. Nicca, J. Notter, P. Paioni, G. Pantaleo, M. Perreau, A. Rauch (Chairman of the Scientific Board), L. Salazar-Vizcaya, P. Schmid, R. Speck, M. Stöckle (Chairman of the Clinical and Laboratory Committee), P. Tarr, A. Trkola, G. Wandeler, M. Weisser, and S. Yerly.

## Data Availability

The datasets presented in this article are not readily available. A request for data sharing can be sent to the Scientific Board of the Swiss HIV Cohort Study. A detailed explanation of the purpose for the request, as well as a study protocol, if applicable, should be presented. The final decision about data release will be taken by the Scientific Board of the SHCS. Requests to access the datasets should be directed to https://www.shcs.ch/.
