# Supplementary material for: Population pharmacokinetics of rilpivirine following oral administration and long-acting intramuscular injection in real-world people with HIV
Source: Front Pharmacol. 2024 Nov 15;15:1437400. doi: 10.3389/fphar.2024.1437400 (PMC11605395; doi:10.3389/fphar.2024.1437400)
Supplement: Supplementary file 1 [file DataSheet1.pdf]

Supplementary Information

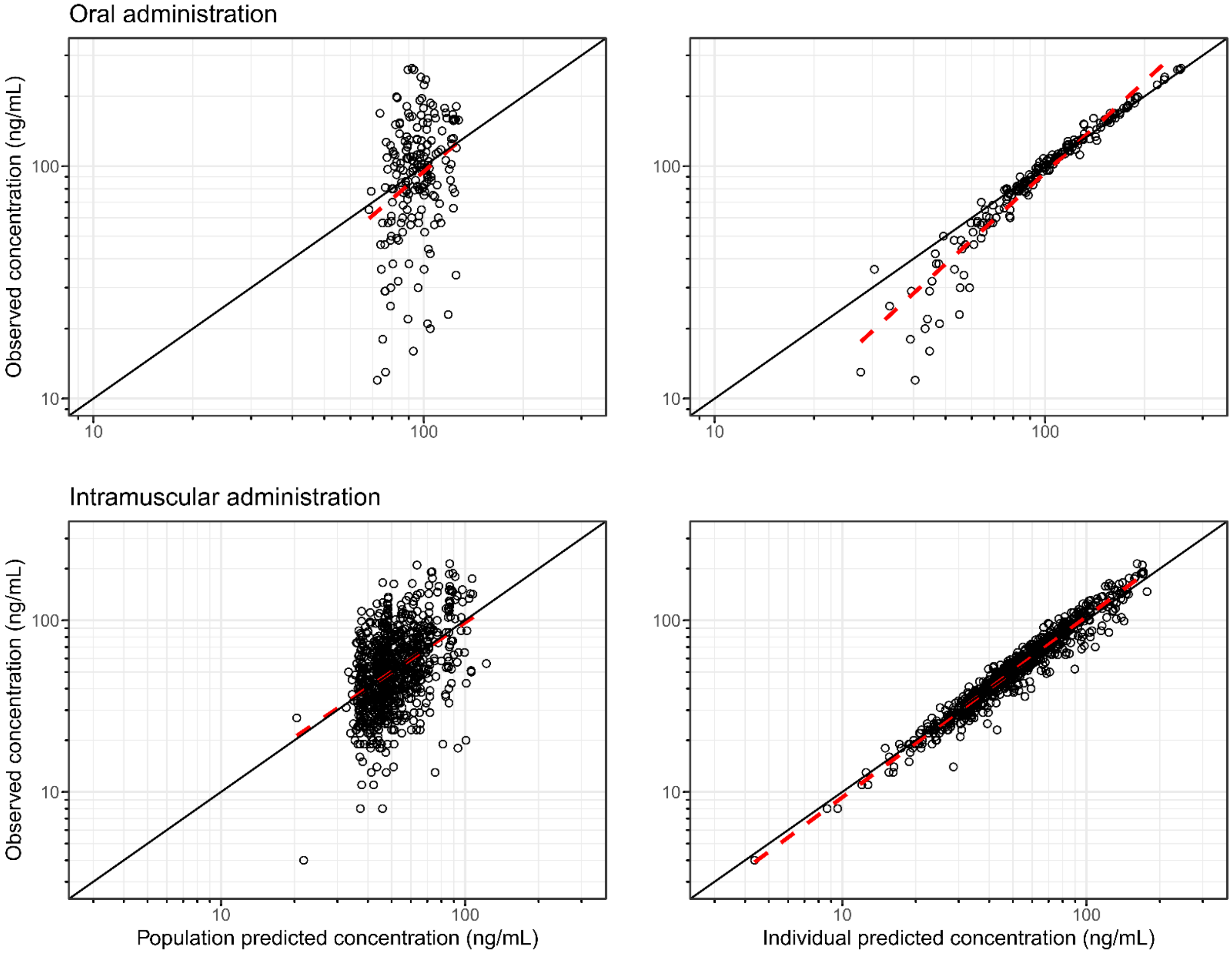

Figure S1: Goodness-of-fit diagnostic plots of the final retained model (without covariate).

Table S1: Results of the cross-validation (n=5) for individual predictions.

Validation	MPE	LLCI 95%	ULCI 95%	RMSE
1	-1.6%	-4.0%	0.8%	19.1%
2	-1.2%	-3.5%	1.1%	17.4%
3	0.4%	-1.5%	2.3%	14.3%
4	-0.7%	-3.0%	1.6%	18.2%
5	-0.5%	-2.3%	1.3%	15.1%
Minimum	-1.6%	-4.0%	0.8%	14.3%
Maximum	0.4%	-1.5%	2.3%	19.1%
Mean	**-0.7%**	**-2.8%**	**1.4%**	**16.8%**

MPE: mean prediction error; LLCI 95%: lower limit of the 95% confidence interval; ULCI 95%: upper limit of the 95% confidence interval; RMSE: root mean-square error.

$$MPE = \frac{\sum C_{obs} - C_{pred}}{N}$$

$$RMSE = \sqrt{\frac{\sum(C_{obs} - C_{pred})^2}{N}}$$

where C_{obs} is the observed log-concentration for the validation subset, C_{pred} is the individual predicted log-concentration, and N corresponds to the number of observations.

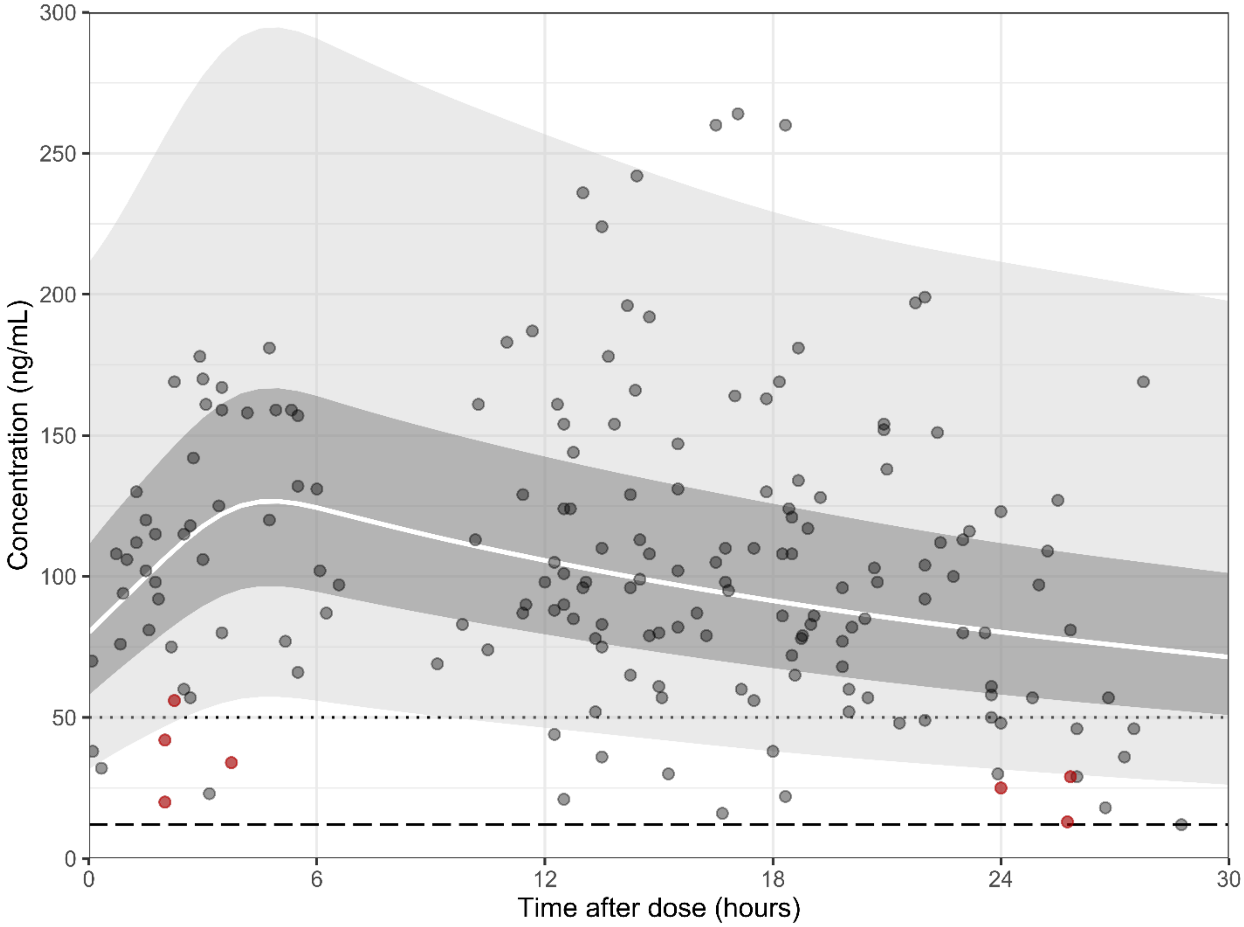

Figure S2: Simulated population percentiles after oral administration of 25 mg of rilpivirine. The solid white line represents the median (50% percentile), while the dark surface encompasses the 50% prediction intervals, and the light surface the 95% prediction intervals. The black dots represent the observed concentrations, while the red dots show the concentrations observed in PWH with gastric bypass. The horizontal dashed line shows the PAIC$_{90}$ of 12 ng/mL, while the dotted line shows the threshold of 50 ng/mL.

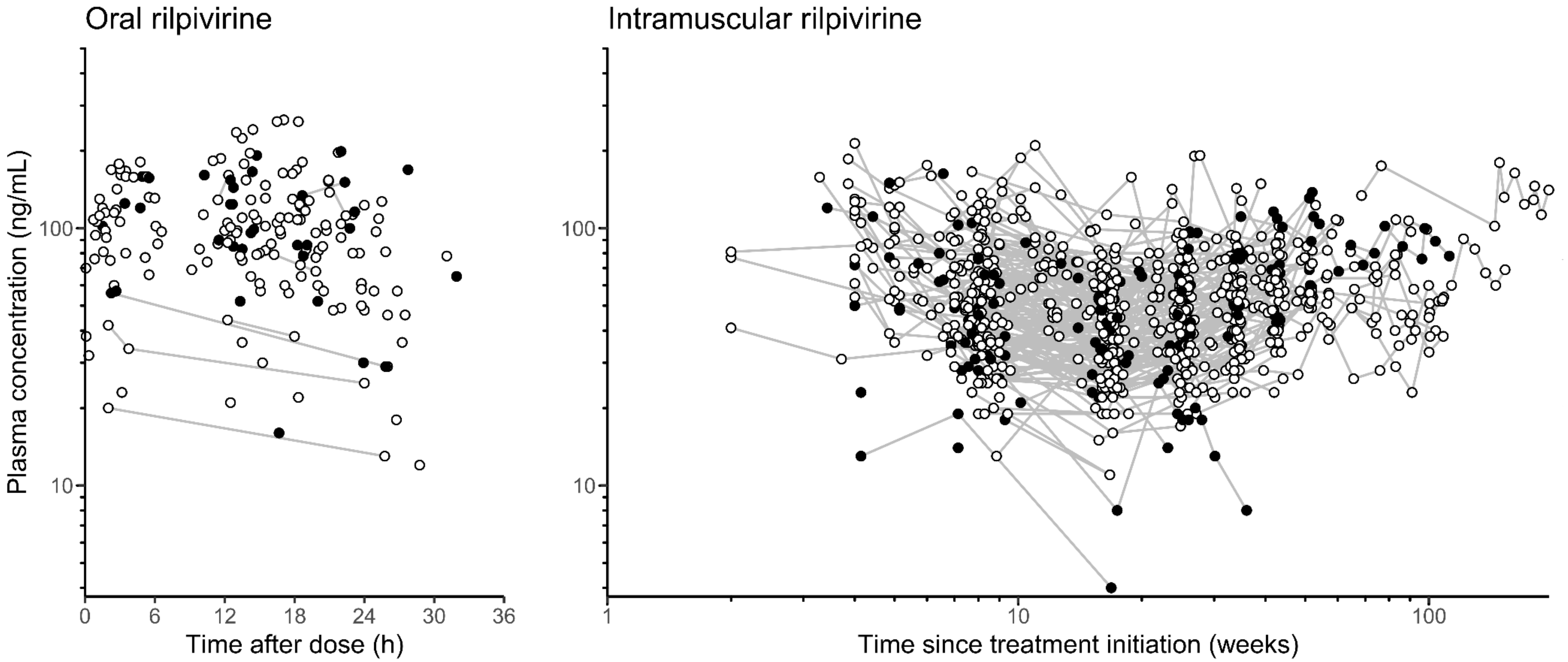

Figure S3: Observed plasma concentrations in females (black circles) and males (empty circles) as a function of time after dose and time since treatment initiation for oral (left panel) and intramuscular (right panel) rilpivirine, respectively. For visual purposes, grey lines connect drug level measurements in the same individuals.

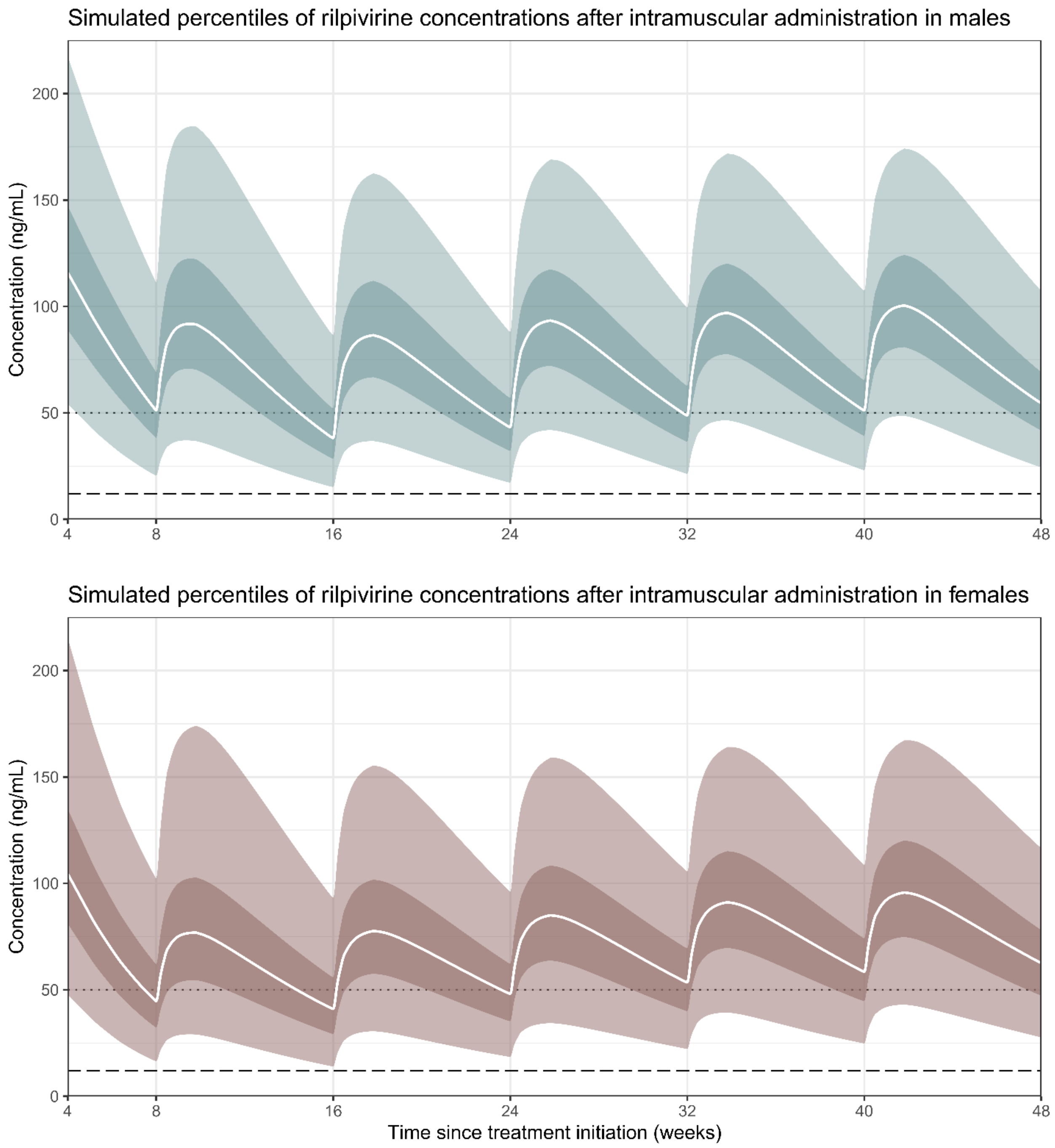

Figure S4: Simulated percentiles stratified by sex after intramuscular administration of rilpivirine following a 4-week period of oral lead-in. The solid white lines represent the median (50% percentile), while the dark surfaces encompass the 50% prediction intervals, and the light surfaces the 95% prediction intervals. The horizontal dashed line represents the PAIC$_{90}$ of 12 ng/mL, while the dotted line shows the threshold of 50 ng/mL.

Table S2: Rilpivirine model-predicted trough concentrations (C_{trough}) over 48 weeks.

Median C_{trough} predictions (ng/mL) [95% prediction intervals]	Week 8	Week 16	Week 24	Week 32	Week 40	Week 48
Males	50 [20 – 109]	38 [15 – 85]	43 [17 – 87]	48 [21 – 98]	51 [23 – 106]	54 [24 – 107]
Females	44 [16 – 101]	41 [14 – 92]	48 [18 – 95]	53 [22 – 105]	58 [25 – 108]	62 [27 – 116]
Model without sex as a covariate	50 [20 – 113]	39 [15 – 89]	45 [18 – 92]	50 [21 – 100]	53 [23 – 110]	55 [26 – 110]

NONMEM CODE FOR FINAL MODEL

```
$INPUT ID DAT1=DROP TIME TIMEAFD AMT DV LAI CMT EVID II SS RATE OCC FEMALE
$DATA      RPV_data.csv     IGNORE=#

$SUBROUTINES ADVAN13 TOL=9
$MODEL
NCOMP=4
        COMP=(DEPOT1)
        COMP=(DEPOT2)
        COMP=(CENTRAL)
        COMP=(PERIPH)

$ABBR REPLACE ETA(OCC)=ETA(5,6,7,8,9,10)

$PK;----------------------------------------------------------------------------------------------------------------------------------------------------
IF (AMT.GT.0) THEN
TDOS=TIME
TAD=0.0
ENDIF
IF (AMT.EQ.0) TAD=(TIME-TDOS)

TVCL = THETA(1)
CL = TVCL * EXP(ETA(1) + ETA(OCC))

V3 = THETA(2)

Q = THETA(3)

V4 = THETA(4)

TVF3 = THETA(5)
F3 = TVF3 * EXP(ETA(2))

TEMP = LOG(THETA(6)/(1-THETA(6)))
```

; If sex is considered as a covariate
; TEMP = LOG(THETA(6)(1+FEMALE*THETA(10))/(1-THETA(6)*(1+FEMALE*THETA(10))))*

```
F1 = EXP(TEMP+ETA(3))/(1+EXP(TEMP+ETA(3)))

F2 = 1 - F1

IF(LAI.EQ.0) THEN
D3 = THETA(7)

ELSE

KA1     = THETA(8)
TVKA2   = THETA(9)
KA2     = TVKA2 * EXP(ETA(4))

ENDIF

K34 = Q/V3
K43 = Q/V4
K30 = CL/V3
S3 = V3/1000
```

```
$DES ;--------------------------------------------------------------------------------------------------------------------------------------------
DADT(1) = -KA1*A(1)
DADT(2) = -KA2*A(2)
DADT(3) = KA1*A(1) + KA2*A(2) - K34*A(3) + K43*A(4) - K30*A(3)
DADT(4) = K34*A(3) - K43*A(4)



$ERROR ;------------------------------------------------------------------------------------------------------------------------------------------
 Q0         = 0
 Q1         = 0

DEL=0
IF (F.EQ.0) DEL=1

 SD1=SQRT(SIGMA(1,1))
 SD2=SQRT(SIGMA(2,2))

 IF (LAI.EQ.0) Q0=1
 IF (LAI.EQ.1) Q1=1

IF (LAI.EQ.0) THEN
 Q0=1
 IPRED0  = F
 W0=SD1
 Y0 = IPRED0+ERR(1)
 IRES0=IPRED0-DV
 IWRES0 = IRES0/W0
ENDIF

IF (LAI.EQ.1) THEN
 Q1=1
 IPRED1  = F
 W1=SQRT((SD2*IPRED1)**2)
 Y1 = IPRED1*(1+ERR(2))
 IRES1=IPRED1-DV
 IWRES1 = IRES1/W1
ENDIF

 IPRED   = IPRED0*Q0+IPRED1*Q1
 Y          = Q0*Y0+Q1*Y1
 IWRES   = Q0*IWRES0+Q1*IWRES1



$THETA ;------------------------------------------------------------------------------------------------------------------------------------------
6.74                    ; CL
277                     ; V3
4.08                    ; Q
839                     ; V4
0.654                   ; F3
0.276                   ; F1
4 FIX                   ; D2 PO

0.00214                 ; KA1 LAI
0.000229                ; KA2 LAI

; if sex is considered as a covariate
; -0.456                ; FEMALE F1
```

```
$OMEGA ;-----------------------------------------------------------------------------------------------------------------------------------------------
0.0649              ; IIV CL
0.129               ; IIV F3
0.708               ; IIV F1
0.521               ; IIV KA2 LAI

$OMEGA BLOCK(1) 0.0167 ; IOV CL
$OMEGA BLOCK(1) SAME
$OMEGA BLOCK(1) SAME
$OMEGA BLOCK(1) SAME
$OMEGA BLOCK(1) SAME
$OMEGA BLOCK(1) SAME


$SIGMA ;-------------------------------------------------------------------------------------------------------------------------------------------------
317                 ; Add PO
0.031               ; Prop LAI

;--------------------------------------------------------------------------------------------------------------------------------------------------------------
$EST METHOD=1 INTER MAXEVAL=2000 NOABORT PRINT=5 SIGL=9 NSIG=3
$COV MATRIX=S
```
